# Characterisation of the vascular pathology in *Sigmodon hispidus* (Rodentia: Cricetidae) following experimental infection with *Angiostrongylus costaricensis* (Nematoda: Metastrongylidae)

**DOI:** 10.1590/0074-02760160124

**Published:** 2017-04-06

**Authors:** Danielle Ingrid Bezerra de Vasconcelos, Ester Maria Mota, Marcelo Pelajo-Machado

**Affiliations:** Fundação Oswaldo CruzFundação Oswaldo CruzInstituto Oswaldo CruzLaboratório de PatologiaRio de JaneiroRJBrasilFundação Oswaldo Cruz-Fiocruz, Instituto Oswaldo Cruz, Laboratório de Patologia, Rio de Janeiro, RJ, Brasil

**Keywords:** Angiostrongylus costaricensis, abdominal angiostrongyliasis, sigmodontinae, arteritis, atherosclerosis

## Abstract

**BACKGROUND:**

*Angiostrongylus costaricensis* is a nematode that causes human abdominal angiostrongyliasis, a disease found mainly in Latin American countries and particularly in Brazil and Costa Rica*.* Its life cycle involves exploitation of both invertebrate and vertebrate hosts. Its natural reservoir is a vertebrate host, the cotton rat *Sigmodon hispidus*. The adult worms live in the ileo-colic branches of the upper mesenteric artery of *S. hispidus*, causing periarteritis. However, there is a lack of data on the development of vasculitis in the course of infection.

**OBJECTIVE:**

To describe the histopathology of vascular lesions in *S. hispidus* following infection with *A. costaricensis.*

**METHODS:**

Twenty-one *S. hispidus* were euthanised at 30, 50, 90 and 114 days post-infection (dpi), and guts and mesentery (including the cecal artery) were collected. Tissues were fixed in Carson’s Millonig formalin, histologically processed for paraffin embedding, sectioned with a rotary microtome, and stained with hematoxylin-eosin, resorcin-fuchsin, Perls, Sirius Red (pH = 10.2), Congo Red, and Azan trichrome for brightfield microscopy analysis.

**FINDINGS:**

At 30 and 50 dpi, live eggs and larvae were present inside the *vasa vasorum* of the cecal artery, leading to eosinophil infiltrates throughout the vessel adventitia and promoting centripetal vasculitis with disruption of the elastic layers. Disease severity increased at 90 and 114 dpi, when many worms had died and the intensity of the vascular lesions was greatest, with intimal alterations, thrombus formation, iron accumulation, and atherosclerosis.

**CONCLUSION:**

In addition to abdominal angiostrongyliasis, our data suggest that this model could be very useful for autoimune vasculitis and atherosclerosis studies.

*Angiostrongylus costaricensis* is a nematode that lives in the ileo-colic branches of the upper mesenteric artery within vertebrate hosts. This parasite causes abdominal angiostrongyliasis, a disease that affects children and adults at similar frequencies (Morera & Céspedes 1971) and is mainly found in Latin American countries, particularly Brazil and Costa Rica.

The parasite’s life cycle includes an intermediate host, usually a slug from the family Veronicellidae (Morera 1973), and a definitive mammalian host, mainly *Sigmodon hispidus* (Morera & Ash 1970). Because humans fail to eliminate parasitic larvae in their faeces (hampering diagnosis of the disease), they are considered accidental hosts (Morera & Céspedes 1971).

Human symptoms range from mild to acute abdominal pain, which may be accompanied by severe intestinal lesions, thickening of the intestinal wall, and an ischemic congestion that is always associated with eosinophilia (Céspedes et al. 1967, Graeff-Teixeira et al. 1991). These common signs can lead to misdiagnosis of other diseases, including colon cancer.

Confirmation of diagnosis requires that three histopathological features be observed: massive infiltration of eosinophils in all layers of the intestinal wall; presence of granulomatous reactions; and eosinophilic vasculitis affecting arteries, veins, lymphatics, and capillaries (Graeff-Teixeira et al. 1991).

Abdominal angiostrongyliasis is an important helminthic disease that affects humans. However, few studies to date have explored the vascular pathology associated with this disease in experimental models.

Our group has been using the cricetid *S. hispidus* as an experimental model for *A. costaricensis* infection because it is its main definitive host in nature, is well adapted to laboratory environments, resists infection better than mice, and shows pathology similar to that of humans (Mota & Lenzi 2005). Particularly at early stages of infection, we have previously observed eggs and larvae inside of capillaries of the intestinal wall and mesentery, as well as in the *vasa vasorum* (VV) of some arteries (Mota & Lenzi 2005). However, there is still a lack of data on the development of eosinophilic vasculitis during infection.

Thus, the purpose of this study was to describe the histopathology of vascular lesions in *S. hispidus* infected with *A. costaricensis*.

## MATERIALS AND METHODS

*Parasite and animals* - The experimental cycle was maintained using *Biomphalaria glabrata* (Say, 1818) molluscs as intermediate hosts. The molluscs were infected (1200 L1/mollusc) with *A. costaricensis* (Crissiumal strain, Rio Grande do Sul, Brazil). L3 parasites were obtained from molluscs at 30 days post-infection (dpi) and delivered to 21 *S. hispidus* via oral gavage (50 L3/animal). Six approximately same-aged non-infected rodents were used as negative controls.

*Ethics* - *S. hispidus* (from the family Cricetidae) were bought from Vyrion Systems (USA) in 1991 and used to establish a colony that has been maintained to present in the Laboratory of Pathology, Oswaldo Cruz Institute, Fiocruz, Rio de Janeiro, Brazil (license IBAMA 34095). Animals were kept in a temperature-controlled room (approximately 21/22ºC) and fed *ad libitium*. All experimental procedures were performed in accordance with the ethical recommendations of the Animal Ethics Committee of the Oswaldo Cruz Foundation (CEUA/ Fiocruz) (licenses LW 43/13 and LW 26/15).

*Histological processing* - Animals were euthanised with a 1:1 mixture of ketamine (10%) and xylazine (2%) at days 30, 50, 90 (five animals/point), and 114 dpi (six animals). The gut and mesentery, including the cecal artery, were collected and fixed in Carson’s Millonig formalin (pH 7.2-7.4) (Carson et al. 1973).

Organs were histologically processed and cut into sections of approximately 5 μm that were subsequently stained with hematoxylin and eosin, Sirius Red at pH 10.2 (for eosinophils) (Bogomoletz 1980, Luque & Montes 1989), Azan trichrome, Perls, Congo Red, and resorcin-fuchsin.

All slides were analysed using a brightfield microscope (Zeiss Axioskop) equipped with a digital camera (Zeiss Axiocam HRc).

## RESULTS

**Fig. 1:**
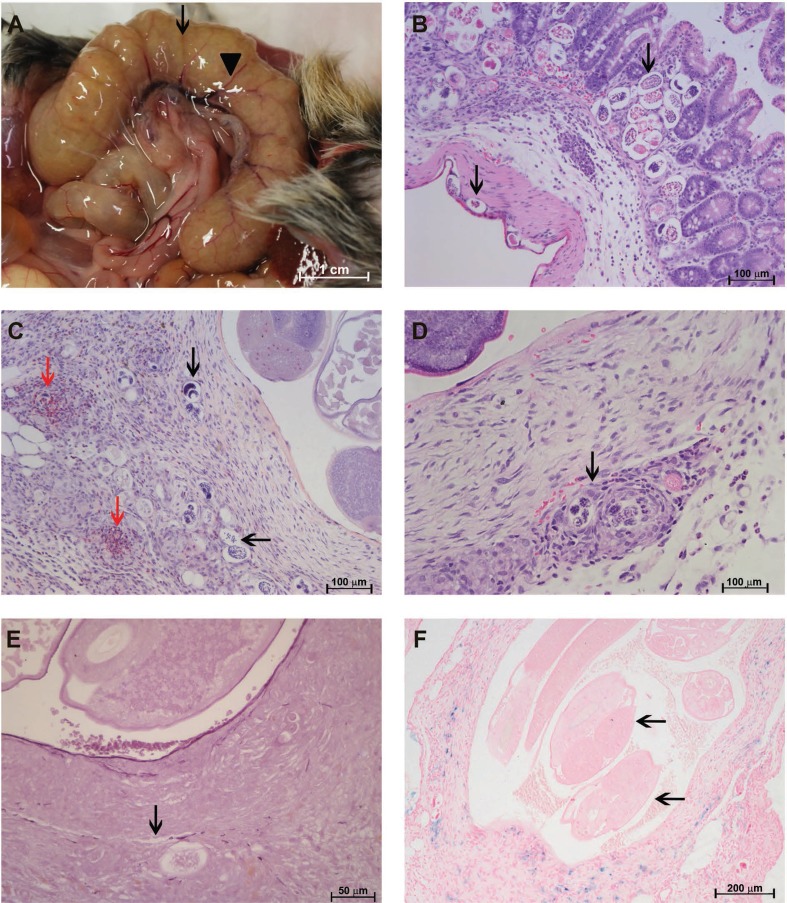
: characterisation of the *Sigmodon hispidus* gut infected with *Angiostrongylus costaricensis* (30 days post-infection). (A) Cecal oedema, accumulation of fluid (arrow), and cecal vessel congestion (arrowhead); (B) eggs and larvae in all gut layers (arrows) (hematoxylin and eosin, HE); (C) eggs and larvae in the *vasa vasorum* of the cecal artery (black arrows) associated with eosinophil infiltrates (red arrows) (HE); (D) granuloma around eggs and larvae in the cecal artery (arrow) (HE); (E) destruction of the external elastic lamina of the cecal artery (arrow) (resorcin-fuchsin); (F) cecal artery with live adult worms in the lumen (arrows) and iron-containing cells along its wall (labelled in blue) (Perls).

The most affected regions of the guts and mesentery during the ovular phase (30 dpi) were the cecum and adjacent mesenterium. The cecum showed an accumulation of fluids and mild congestion, whereas the cecal artery was darkened and enlarged (Fig. 1A). Embryonated eggs and larvae were found in all layers of the gut via brightfield microscopy (Fig. 1B).

The VV of the cecal artery, a niche for adult worms, had many larvae and eggs and an intense infiltrate composed mainly of eosinophils that formed confluent granulomas (Fig. 1D). These parasitic elements and cellular infiltrates were associated with destruction of the external elastic lamina of the artery wall (Fig. 1E).

Live male and female adult worms with mild iron accumulation in their walls were often found inside the cecal artery (Fig. 1C-F).

**Fig. 2:**
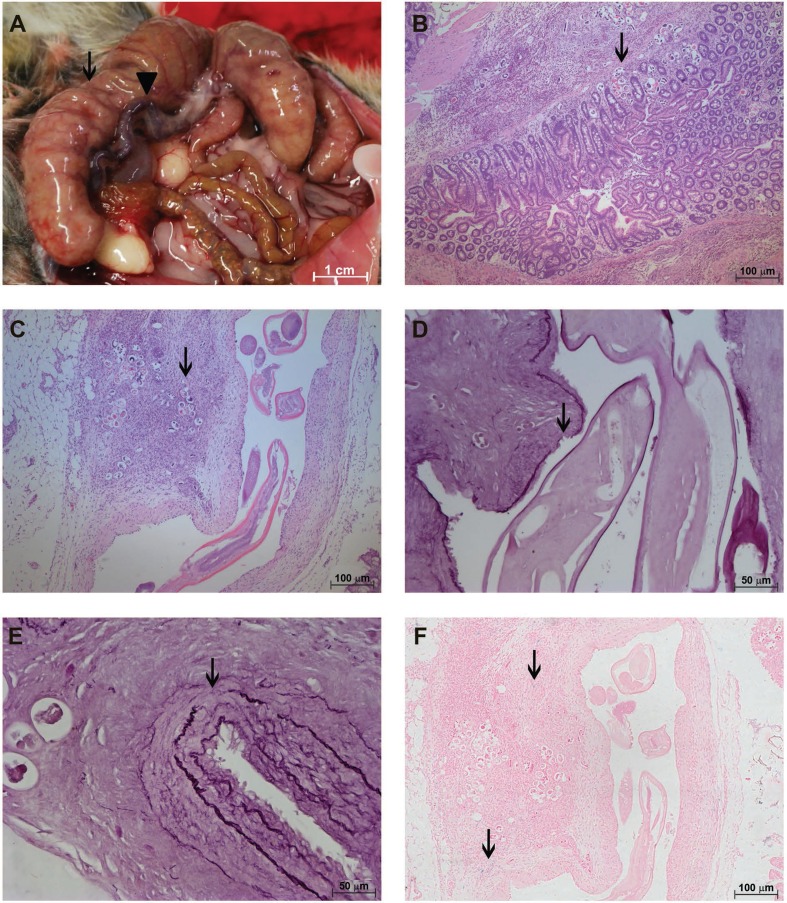
: characterisation of the *Sigmodon hispidus* gut infected with *Angiostrongylus costaricensis* (50 days post-infection). (A) Cecum with signs of sufferance (arrow) and cecal artery congestion (arrowhead); (B) eggs and larvae in the gut (arrow) (hematoxylin and eosin, HE); (C) eggs and larvae (arrow) in the *vasa vasorum* of the cecal artery together with inflammatory infiltrates causing protrusions into the media and intima layers inside the vessel (HE); (D) destruction of the external and internal elastic fibres (arrow) of the cecal artery with parasites (resorcin-fuchsin); (E) destruction of the external elastic layer of the cecal artery without parasites in the segment (arrow) (resorcin-fuchsin); (F) small amount of iron around the cecal artery with live parasites (arrows) (Perls).

The cecum was darkened and corrugated at 50 dpi, indicating sufferance of this intestinal segment (Fig. 2A).

Histological analysis revealed the presence of eggs and larvae in all layers of the gut (Fig. 2B). Together with inflammatory infiltrates, these parasitic elements in the VV caused thickening of the adventitia of the cecal artery, occasionally causing the vessel wall to protrude into the arterial lumen (Fig. 2C). The latter was frequently accompanied by muscular destruction and/or discontinuity of the internal and external elastic layers (Fig. 2D), including artery segments that did not contain parasites (Fig. 2E).

Similar to what was observed at 30 dpi, live adult worms with a small amount of accumulated iron were found inside the cecal arteries at 50 dpi (Fig. 2F).

**Fig. 3:**
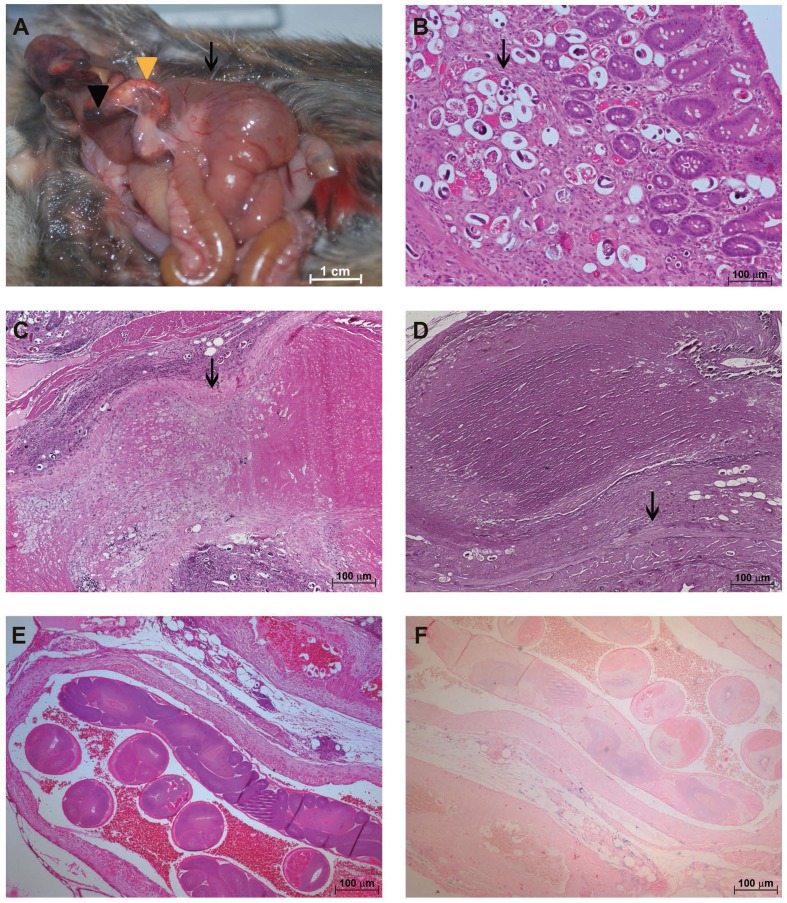
: characterisation of the *Sigmodon hispidus* gut infected with *Angiostrongylus costaricensis* (90 days post-infection). (A) Cecal oedema (arrow) and cecal artery congestion composed of a clot (black arrowhead) and yellow substance (yellow arrowhead); (B) eggs and larvae inside small vessels (arrow) (hematoxylin and eosin, HE); (C) intimal destruction with eggs, larvae, and inflammatory infiltrates in the *vasa vasorum* of the cecal artery (arrow) (HE); (D) destruction of the external and internal elastic fibres of the cecal artery containing a thrombus (arrow) (resorcin-fuchsin); (E) cecal artery containing adult parasites (HE); (F) cecal artery containing live adult parasites and no remarkable deposits of iron along its wall (Perls).

Disease in these two time points (30 and 50 dpi) was less severe than at later stages of infection (90 and 114 dpi). At the macroscopic level, the ceca from rodents at 90 dpi were strongly distended because of the accumulation of air and fluid, and the cecal arteries were almost completely congested by thrombi and a yellow substance (Fig. 3A).

As in earlier stages of infection, eggs and larvae were present in all intestinal layers of animals at 90 dpi. However, at this stage, many eggs were unembryonated and calcified (Fig. 3B).

The middle layer of the cecal artery was invaded by an inflammatory infiltrate that associated with fibrin thrombi, leading to disappearance of the intimal layer (Fig. 3C). At the same time, the internal and external elastic laminas were completely destroyed (Fig. 3D).

**Fig. 4:**
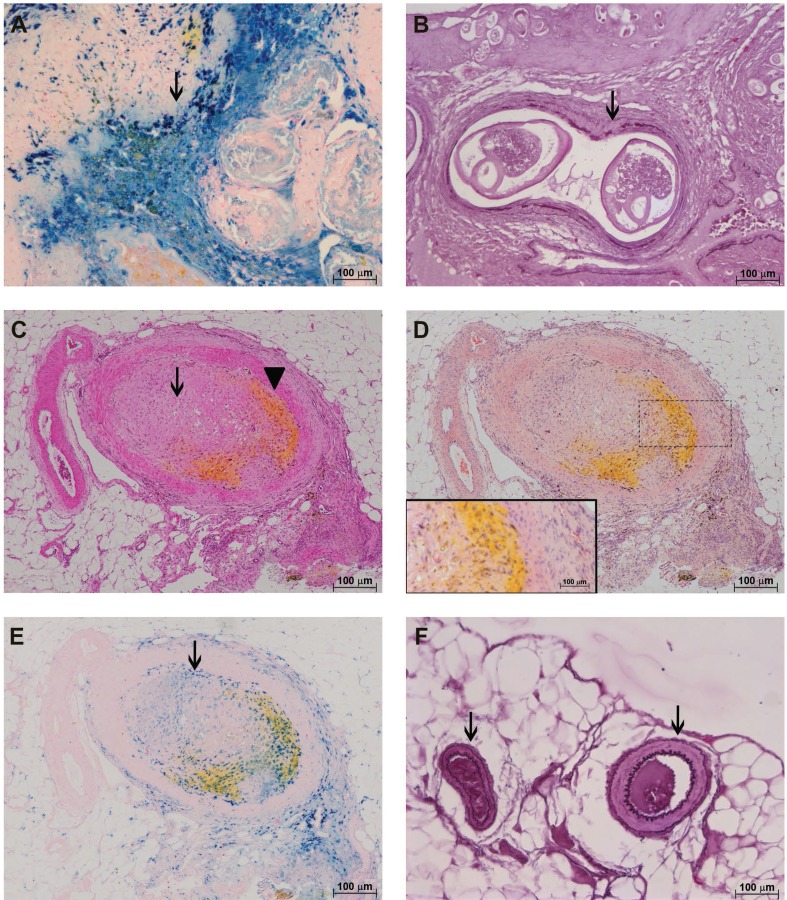
: characterisation of the *Sigmodon hispidus* gut upon infection with *Angiostrongylus costaricensis* (90 days post-infection). (A) Cecal artery containing dead adult parasites and accumulated iron (arrow) (Perls); (B) destruction of the external and internal elastic fibres of the cecal artery (arrow) (resorcin-fuchsin); (C) occlusion of the mesenteric vessel by a thrombus (arrow) and yellow substance (arrowhead) (hematoxylin and eosin); (D) occlusion of the mesenteric vessel by a thrombus and yellow substance not accompanied by an eosinophil infiltrate (Sirius Red, pH = 10.2); (E) occlusion of the mesenteric vessel by a thrombus and yellow substance associated with iron (arrow) (Perls); (F) mesenteric vessels with normal morphology (arrows) (resorcin-fuchsin).

A considerable amount of live and dead adult parasites was found in the cecal artery (Fig. 3E). Iron deposits, found in discreet foci in living worms (Fig. 3F), were distributed throughout the dead worms (Fig. 4A). Arteries containing worms showed almost complete destruction of the external elastic lamina and an important disruption of the internal layer (Fig. 4B).

Some segments of mesenteric arteries were obliterated. These had a yellow substance in their intimal layers (Fig. 4C) without eosinophilic infiltrates (Fig. 4D), and they accumulated iron (Fig. 4E). Other segments appeared normal, with intact elastic laminas (Fig. 4F).

**Fig. 5:**
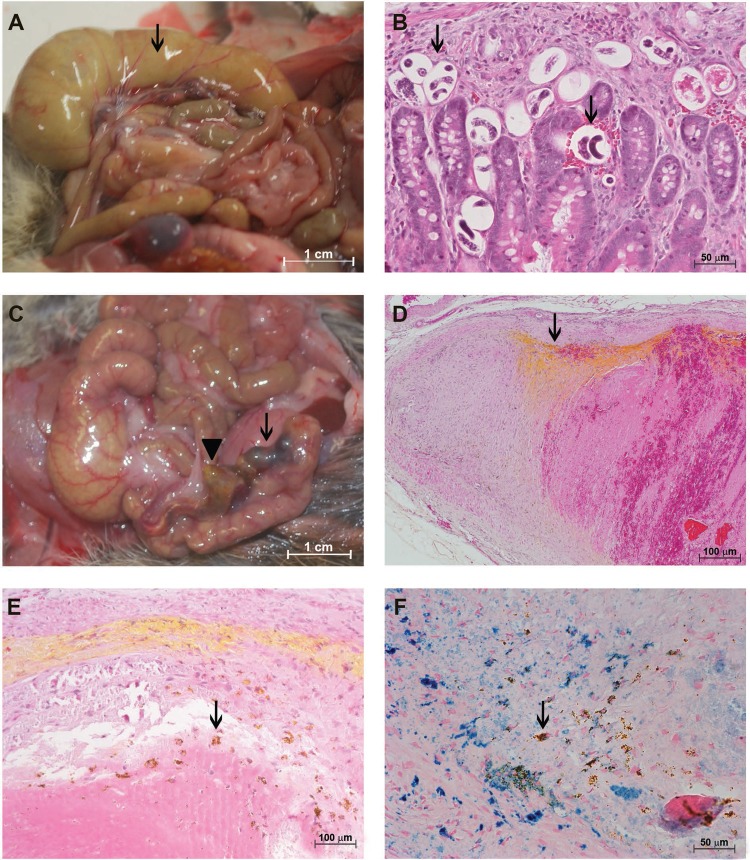
: characterisation of the *Sigmodon hispidus* gut upon infection with *Angiostrongylus costaricensis* (114 days post-infection). (A) Cecal oedema, accumulation of fluid (arrow), and congestion of cecal vessels; (B) eggs and larvae in the gut (arrows) (hematoxylin and eosin, HE); (C) cecal artery congestion by a clot (arrow) and yellow substance (arrowhead); (D) thrombus with yellow substance in lamellar aspect (arrow) (HE); (E) thrombus with macrophages carrying a yellow substance (arrow) (HE); (F) thrombus with macrophages carrying a yellow substance associated with iron (arrow) (Perls).

At the latest time point of infection (114 dpi), fluid and air accumulation in ceca was accompanied by congestion of cecal vessels (Fig. 5A). As before, all intestinal layers were parasitised, containing either embryonated or dead eggs (Fig. 5B).

**Fig. 6:**
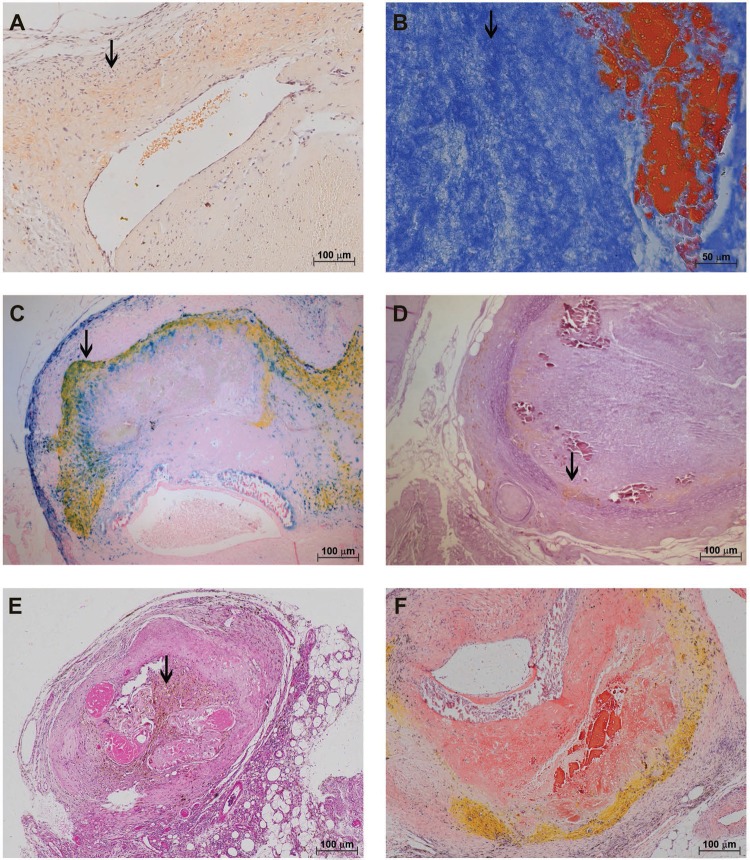
: characterisation of the *Sigmodon hispidus* gut upon infection with *Angiostrongylus costaricensis* (114 days post-infection). (A) Yellow substance labelled with Congo’s Red (red arrow); (B) a thrombus is composed primarily of fibrin (arrow) (Azan); (C) thrombus with a yellow substance associated with a considerable amount of iron (arrow) (Perls); (D) destruction of the external and internal elastic layers of the cecal artery with a thrombus (arrow) (resorcin-fuchsin); (E) cecal artery containing adult parasites and macrophages carrying a yellow substance (arrow) (hematoxylin and eosin); (F) thrombus in the cecal artery containing a yellow substance and lacking eosinophil infiltrates (Sirius Red, pH = 10.2).

Segmental involvement of the cecal artery was evident (even to the naked eye), with extensive obstruction of the vessel by large thrombi and yellowish deposits (Fig. 5C). These deposits formed a lamellar pattern mostly in the intimal layer (Fig. 5D-E) and were carried by cells that were most likely macrophages. Perls reaction (Fig. 5F) showed that within a population of iron-containing cells, some also carried this xanthomatous substance (labelled by Congo Red, Fig. 6A, but not apparent in polarised light, not shown). Thrombi were mostly composed of fibrin (Fig. 6B) and a significant amount of iron (Fig. 6C). Furthermore, muscular disorganisation of cecal arteries were observed at this last time point (Fig. 6D).

In cecal arteries containing dead parasites, phagocytic cells carrying the aforementioned yellowish substance were also abundant (Fig. 6E). Inflammation associated with the thrombus did not contain eosinophils (Fig. 6F).

**Fig. 7:**
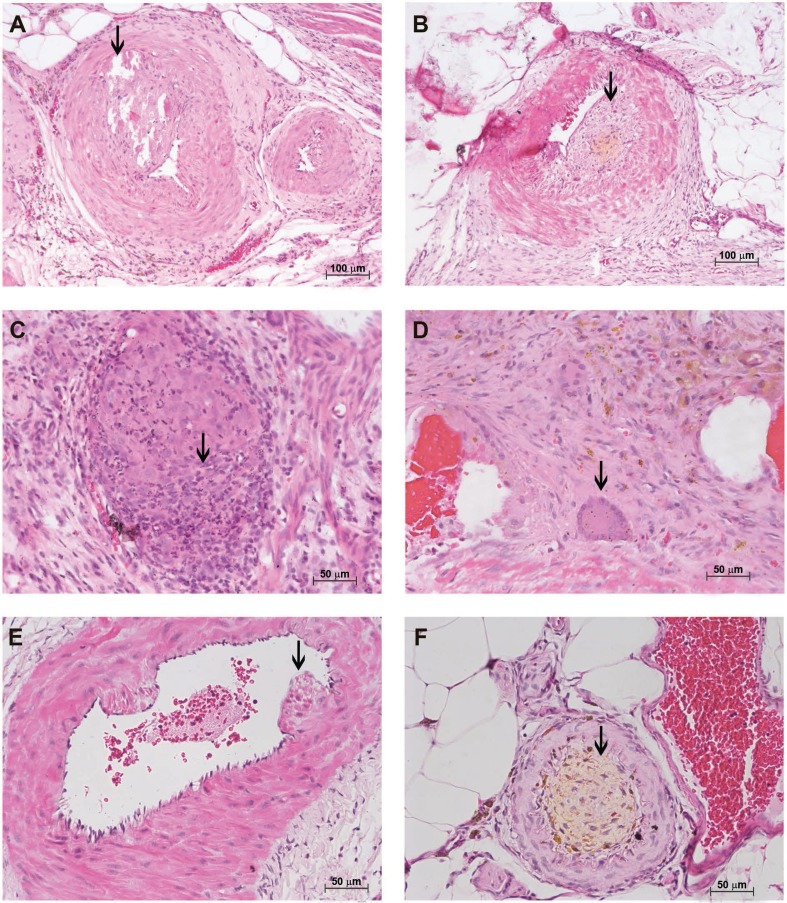
: characterisation of the *Sigmodon hispidus* upon infection with *Angiostrongylus costaricensis* (114 days post-infection). (A) Thrombus recanalisation (arrow) (hematoxylin and eosin, HE); (B) thickening of the intima with yellow deposit (arrow) (HE); (C) vessel obliterated with inflammatory infiltrate (arrow) (HE); (D) giant cell (red arrow) (HE); (E) pseudopolyp in the mesenteric vessel (arrow) (HE); (F) mesenteric vessel blocked with cells carrying a yellow substance (arrow) (HE).

Different features indicated vascular involvement at 114 dpi: thrombus recanalisation (Fig. 7A); intimal thickening with yellowish lesions (Fig. 7B); obliterated vessels with an inflammatory infiltrate rich in dead neutrophils (Fig. 7C); the presence of giant cells (Fig. 7D); pseudopolyps in the mesenteric vessel (Fig. 7E); and blockage of the mesenteric vessel by cells carrying xanthomatous material (Fig. 7F).

Table summarises microscopic findings in infected animals at the various time points. The control animals showed no parasites or histological abnormalities.

**TABLE:** Summary of microscopic findings in infected animals at various time points. The control rodents showed no histological abnormalities

Time of infection	Microscopic findings
30 dpi	Frequent occurrence of live adult male and female worms inside the cecal artery. Presence of numerous embryonated eggs and larvae in all layers of the gut and in the *vasa vasorum* (VV) of the cecal artery. Eosinophil-rich confluent granulomas around alive eggs and larvae. Cecal artery’s external elastic lamina often damaged. Mild deposit of Perls-revealed iron along the wall of worm-containing arteries.
50 dpi	Frequent occurrence of live adult male and female worms inside the cecal artery. Presence of numerous embryonated eggs and larvae in all layers of the gut and in the VV of the cecal artery. Eosinophil-rich confluent granulomas around alive eggs and larvae. Adventitial thickening, often accompanied by muscular layer partial destruction. Cecal artery’s external and internal elastic laminas often damaged. Occasional protrusion of the arterial wall towards the lumen. Mild deposit of Perls-revealed iron along the wall of worm-containing arteries.
90 dpi	Occurrence of live and dead adult worms inside the cecal artery. The dead ones showed globally-distributed iron accumulation. Many eggs in the intestinal layers and in the arteries walls were unembryonated, calcified and showed iron deposition. Eosinophil infiltration restricted to alive eggs and larvae. Thrombosis of the cecal artery, with disappearance of its intimal layer and intense inflammatory infiltration of the muscular one. Complete destruction of the cecal artery’s external and internal elastic laminas. Obliteration of segments of cecal and mesenteric arteries with xantomatous substance accumulation in their intimal layers.
114 dpi	Most adult worms inside the cecal artery were dead and showed iron accumulation. Many eggs in the intestinal layers and in the arteries walls were unembryonated, calcified and showed iron deposition. Eosinophil infiltration reduction, restricted to alive eggs and larvae. Extensive obstruction of the cecal artery, combining large thrombi and yellowish deposits (atherosclerosis), often associated with the presence of macrophages containing this yellow substance, sometimes together with iron. Disorganisation of the muscular layer of the cecal artery Complete destruction of the cecal artery’s external and internal elastic laminas. Eventual recanalisation of the observed thrombi. Presence of projections of the intimal layer towards the arterial lumen, in a pseudopolypous-like pattern. Obstruction of some mesenteric vessels by clots of xantomatous substance-containing cells.

## DISCUSSION

In this study, eggs and larvae trapped in the VV of big arteries of the cecal region, accompanied by mononuclear and eosinophilic cells, was evident at all stages of infection analysed. In early stages of infection (30 and 50 dpi), most of the parasites (eggs and larvae) were alive and the infiltrate did not seem to destroy them. On the other hand, at later stages (90 and 114 dpi), many immature and degenerate (somewhat calcified) eggs were observed, consistent with the presence of dead adult worms inside the arteries. These dead eggs did not appear to be associated with eosinophils in the inflammatory response, but rather with macrophages. This suggests that only live eggs and L1 larvae cause intense eosinophil infiltration, by promoting the release of chemical factors, possibly IL-5 and/or eotaxin, that attract the cells.

The inflammatory infiltrate begins at the adventitial layer and extends toward the intima, reducing the arterial lumen. During this process, inflammatory cells invade the muscular layer after damaging the external elastic lamina, passing through the muscular cells and resulting in centripetal arteritis. This pattern suggests that alterations in the intima could be secondary to this centripetal gradient of antigens and inflammation. However, without excluding this first mechanism, it is also possible that some endothelial damage is caused directly by adult worms, particularly dead worms and substances they excrete. In fact, the death of *A. costaricensis* worms seems to be a turning point in the course of infection. These and other intravascular helminths are successfully adapted to this environment, controlling homeostasis, for example, releasing anti-coagulant molecules (Ranasinghe et al. 2015). Starting at 90 dpi, when the adventitial lesions were larger and adult worms had died, we observed the development of thrombi and an atherogenic-like process. This process was characterised by the deposition of xanthomatous material and presence of macrophages, features compatible with so-called “foam cells”.

Some chronic inflammatory diseases, including rheumatic pathologies, also end with the development of atherosclerosis. Evidence shows that autoimmune vasculitis is the major mechanism responsible for the accelerated atherogenesis of rheumatoid arthritis, Takayasu’s arteritis, and systemic lupus erythematosus (Bacon et al. 2002, Rav-Acha et al. 2007).

Eotaxin and its receptor (CCR3) are overexpressed in human atherosclerosis and contribute to vascular inflammation (Haley et al. 2000). In a study using ApoE knockout (KO) mice, administration of anti-eotaxin-2 polyclonal blocking antibodies was shown to reduce early atheroma. When administered continuously, it promoted stabilisation of atheroma (Mor et al. 2013). These findings suggest the possibility that eosinophil chemoattractive proteins, which can be delivered either by inflammatory cells or by parasitic elements, are involved in the physiopathology of atherogenesis.

Foam cells (macrophages with lipids) aid in the development of an initial lesion. Once this lesion is sustained, the arterial changes become irreversible, resulting in smooth muscle cell migration and proliferation, fibrous tissue matrix deposition, calcification, angiogenesis, and necrotic core formation (Bentzon et al. 2014). In the current study, we observed these macrophage foam cells, some of which contained iron deposits. However, it was not clear whether they played a role in the atherogenic-like process or if they acted as scavenger cells to clean up the deposits. We cannot be certain of the nature of the content of these cells. Nevertheless, we assume that the xanthomatous substance is a lipid or lipoprotein complex, and possibly a product of deteriorating worms or a metabolic expression of inflammation after initiation of an endothelium lesion.

Iron has also been implicated in this process. Some studies have shown that iron accumulation is related to atherosclerosis (Juckett et al. 1995, Kiechl et al. 1997). The presence of serum ferritin is the strongest risk factor for overall progression of carotid atherosclerosis. The first steps of atherosclerosis are related to the capacity of ferritin to modify the atherogenic potential of low-density lipoprotein (LDL) (Kiechl et al. 1997). The classical atherosclerosis physiopathology involves LDL retention in the arterial intimal, its oxidation, and other types of modification that activate the immune system (Miller et al. 2011). Our model showed intense accumulation of iron inside arteries with thrombi or dead parasites, and within foam cells and other macrophages. Because iron accumulated inside and around non-embryonated eggs, it is possible that it originated within the eggs. With another helminth, *Schistosoma mansoni*, the uptake of heme is necessary for oogenesis (Toh et al. 2015). Therefore, with *A. costaricensis*, it is possible that excess iron in non-embryonated eggs accumulates in arteries and contributes to the development of atherosclerotic lesions.

Reduced elasticity of the cerebral arteries, secondary to disruption of the elastic laminas (particularly the external one), has also been associated with the atherosclerotic process (Masuoka et al. 2010). In our study, destruction of the external elastic layer began at 30 dpi. During infection, the internal layer was also destroyed, causing complete disorganisation of the cecal artery structure, rendering it more rigid and susceptible to atherosclerosis. A possible mechanism underlying disruption of the elastic laminas is the production of metalloproteinase 12 by eosinophils in the adventitial infiltrate, as previously shown to occur in meningitis caused by *Angiostrongylus cantonensis* (Wei et al. 2011).

Studies of atherosclerosis have several limitations, particularly the scarcity of experimental models. Although it is inexpensive to maintain and breed wild-type mice, these mice do not express cholesterol ester transfer protein, a plasma protein putatively involved in human atherogenesis (Getz & Reardon 2012).

An alternative solution would be to use KO mice. The ApoE KO mouse is the most popular model because it develops atheromas on a standard chow diet and shows disease similar to that in humans. This model does not have a glycoprotein that participates in cholesterol clearance and homeostasis. Atherosclerotic lesions (with the presence of foam cells) occur by week 10, and the presence of fibrous plaques in the aorta, coronary, and pulmonary arteries is evident by week 20 (Plump et al. 1992, Zhang et al. 1992). In the LDLR^−/−^ model (familial hypercholesterolemia), development of significant lesions, particularly in older animals, requires feeding a high-fat atherogenic diet (Baract et al. 2006). In addition to KO mouse models, the wild rat model with balloon injury to the carotid artery develops early lesions associated with smooth muscle contraction and late lesions associated with intimal thickness (Clowes et al. 1983).

Surprisingly, while we studied vasculitis in the cecal artery of *S. hispidus* infected with *A. costaricensis*, we observed characteristics of atherosclerosis. It is not clear whether these features should be expected in other models or are specific to infection of *S. hispidus* with *A. costaricensis*. Nevertheless, this model opens a wide range of possibilities to explore the physiopathology of autoimmune vasculitis and atherosclerosis.
